# Supplementary material for: A Critical View of the Application of the APEX Software (Aqueous Photochemistry of Environmentally-Occurring Xenobiotics) to Predict Photoreaction Kinetics in Surface Freshwaters
Source: Molecules. 2019 Dec 18;25(1):9. doi: 10.3390/molecules25010009 (PMC7017383; doi:10.3390/molecules25010009)
Supplement: Supplementary file 1 [file molecules-25-00009-s001.zip › 3.2.4_gcc-4.4.0.zip!/3.2.4_gcc-4.4.0/3.2.4_gcc-4.4.0/doc/octave/html/liboctave/Ranges.html]

Ranges - Octave C++ Classes


Next: Nonlinear Functions,
Previous: Matrix Factorizations,
Up: Top


---

## 6 Ranges

— : **Range** (void)  
— : **Range** (const Range &r)  
— : **Range** (double b, double l)  
— : **Range** (double b, double l, double i)

— : double **base** (void) const  
— : double **limit** (void) const  
— : double **inc** (void) const

— : void **set\_base** (double b)  
— : void **set\_limit** (double l)  
— : void **set\_inc** (double i)

— : int **nelem** (void) const

— : double **min** (void) const  
— : double **max** (void) const

— : void **sort** (void)

— : ostream& **operator <<** (ostream &os, const Range &r)  
— : istream& **operator >>** (istream &is, Range &r)

— : void **print\_range** (void)
